# Feeding ecology and sexual dimorphism in a speciose flower beetle clade (Hopliini: Scarabaeidae)

**DOI:** 10.7717/peerj.4632

**Published:** 2018-06-20

**Authors:** Jonathan F. Colville, Mike D. Picker, Richard M. Cowling

**Affiliations:** 1 Kirstenbosch Research Centre, South African National Biodiversity Institute, Cape Town, South Africa; 2 Statistics in Ecology, Environment and Conservation, University of Cape Town, Rondebosch, South Africa; 3 Department of Biological Sciences, University of Cape Town, Rondebosch, South Africa; 4 African Centre for Coastal Palaeoscience, Nelson Mandela Metropolitan University, Port Elizabeth, South Africa

**Keywords:** Hind legs, Male–male combat, Sexual selection, Monkey beetles, Cape region, Mating systems, Allometry

## Abstract

The relationship between feeding ecology and sexual dimorphism is examined in a speciose South African monkey beetle clade. We test whether feeding and mating at a fixed site (embedding guild) is associated with greater levels of sexual dimorphism and possibly sexual selection than species using unpredictable feeding resources (non-embedding guild). Sexual dimorphism was measured using a point scoring system for hind leg and colour across the two feeding guilds for >50% of the regional fauna. Quantification of hind leg dimorphism using a scoring system and allometric scaling were used to identify traits subject to sexual selection. Feeding guild had a significant effect on hind leg dimorphism, with embedders having high and non-embedders low scores. The sessile and defendable distribution of females on stable platform flowers may favour contests and associated hind leg weaponry. In contrast, degree of colour dimorphism between the sexes was not associated with any particular feeding guild, and may serve to reduce male conflict and combat. Embedder males had high proportions (∼76%) of species with positive allometric slopes for almost all hind leg traits. For male non-embedders, only ∼37% of species showed positive scaling relationships. Phylogenetic data, in conjunction with behavioural data on the function of leg weaponry and visual signalling among males is needed to better understand the link between sexual dimorphism and sexual selection in the radiation of the monkey beetles.

## Introduction

Sexual dimorphism is widespread across many animal groups and is generally considered to be the result of sexual selection (Hedrick & Temeles, 1989; Andersson, 1994; Fairbairn, 1997), although natural selection is also an important mechanism in its evolution (Shine, 1989; Temeles et al., 2000; Cooper, 2010; Svenson et al., 2016). Sexual dimorphism manifests in various forms—including colour differences (Greenwood, 1974; Barraclough, Harvey & Nee, 1995), ornaments and weapons (Emlen, 2008).

The strength of sexual selection appears best understood in the context of mating systems that differ in ecological and environmental factors that influence male–male competition (Emlen & Oring, 1977; Thornhill, 1981; Bjorklund, 1991; Shuster, 2009; Machado et al., 2016). For example, if ecological factors allow for the clustering of a resource (e.g. females or oviposition sites), males evolve mating systems that most economically defend resources from other males (*sensu* resource monopolisation; Emlen & Oring, 1977), and allow for greater male fitness through increased matings and mate guarding opportunities (Thornhill & Alcock, 1983). Under such scenarios, sexual selection is expected to be high. In contrast, ecological factors may disperse females more uniformly, with males evolving mating strategies of increased searching for females (Fincke, Waage & Koenig, 1997) and reduced aggression and mate guarding, i.e. less intense sexual selection. The operational sex ratio within a population also influences the intensity of sexual selection, with male–male competition and defence of resources expected to be high in male-biased populations (Emlen & Oring, 1977). The potential for sexual selection can therefore vary from high to low depending on the relative rarity of females, and one can make predictions about patterns of sexual dimorphism across different ecological factors and mating systems.

The intensity of sexual selection has been linked to both levels of sexual dimorphism, and positive allometric scaling relationships in dimorphic structures (exaggerated traits important in intrasexual contests) (Bonduriansky & Day, 2003; Kodric-Brown, Sibly & Brown, 2006). Exaggerated traits used in male combat which display positive allometric slopes (>1) are mostly indicative of directional sexual selection (Andersson, 1994, Emlen & Nijhout, 2000, Bonduriansky & Day, 2003; Kodric-Brown, Sibly & Brown, 2006; but see Emlen & Nijhout, 2000; Bonduriansky, 2007 for exceptions). The advantages for larger males to have disproportionately larger trait sizes are increased reproductive success and survivorship (Thornhill & Alcock, 1983; Andersson, 1994, Jennions, Moller & Petrie, 2001), with steep allometric slopes reflecting high levels of sexual selection.

Monkey beetles (a tribe of the Scarabaeoidea) provide a suitable taxon for investigating how ecological factors might generate sexual dimorphism. They are very diverse, and have variable feeding ecology and degrees of sexual dimorphism. Approximately 63% of the world’s fauna (∼1,040 species) and 38% of the genera are found in South Africa (Colville et al., 2014). Impressively >50% of these species are concentrated within the relatively small Cape region (∼15% of the country) (Manning & Goldblatt, 2012; Snijman, 2013), the global centre for monkey beetle radiation.

**Figure 1:**
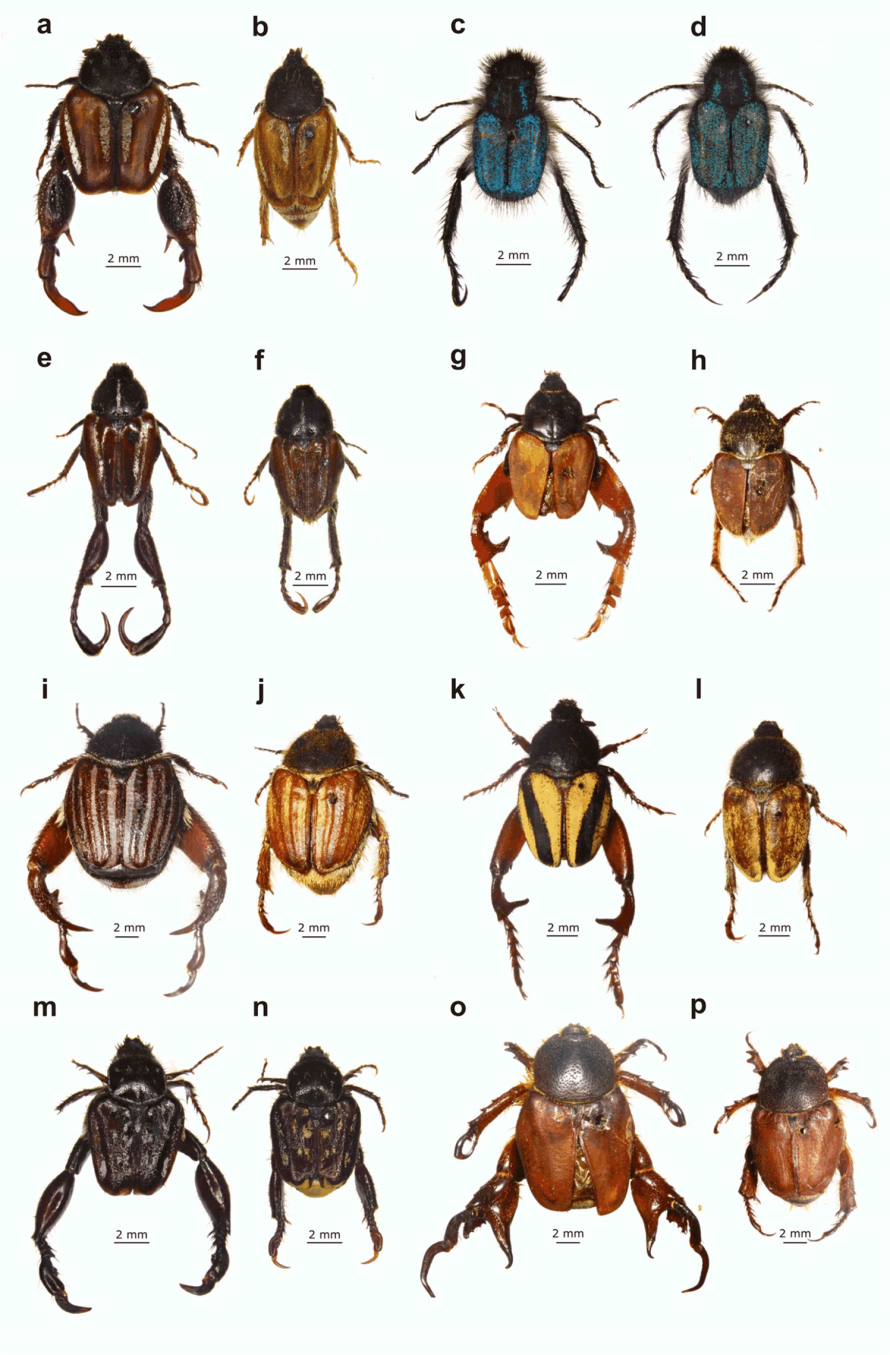
Examples of dimorphic South African monkey beetles showing diversity in body and hind leg shape and form, and colour pattern. Male at left for each pair: (A and B) *Pachycnema calcarata* (Burmeister, 1844), (C and D) *Anisonyx ditus* (Péringuey, 1902), (E and F) *Heterochelus bivittatus* (Burmeister, 1844), (G and H) *Heterochelus detritus* (Burmeister, 1844), (I and J) *Denticnema striata* (Burmeister, 1844), (K and L) *Heterochelus chiragricus* (Thunberg, 1818), (M and N) *Pachycnema crassipes* (Fabricius, 1775), (O and P) *Hoplocnemis crassipes* (Olivier, 1789). (Photo credit: Mike Picker).

**Figure 2:**
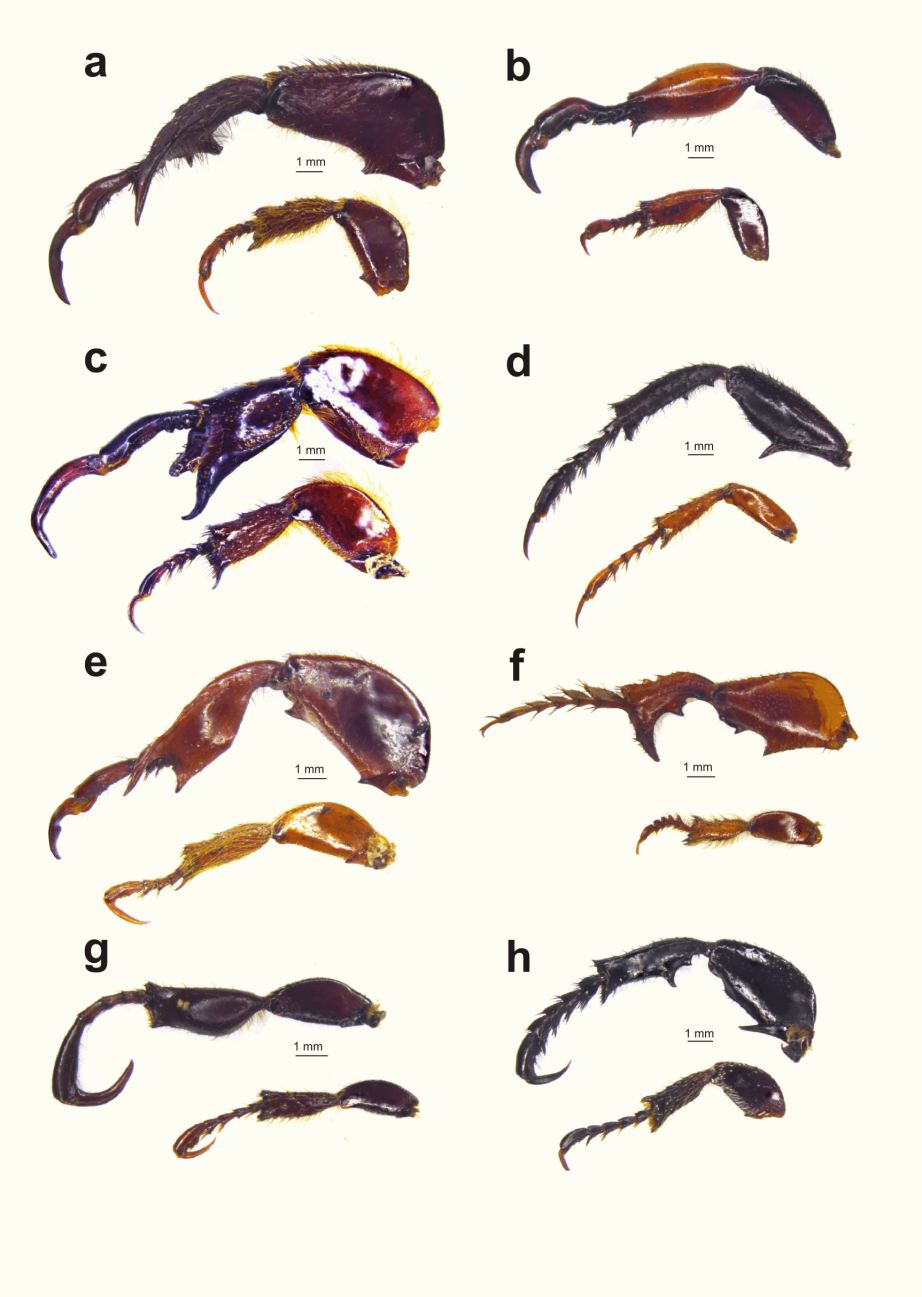
Diversity of male hind leg traits and weaponry for eight embedding monkey beetle species. Diversity of male hind leg traits and weaponry for eight embedding species. Female leg displayed (at same scale) below male leg. (A) *Denticnema striata*, (B) *Pachycnema alternans* (Burmeister, 1844), (C) *Hoplocnemis crassipes*, (D) *Mauromecistoplia nieuwoudtvillensis*, (E) *Denticnema decemlineata* (Dombrow, 1997), (F) *Heterochelus detritus*, (G) *Heterochelus bivittatus*, (H) *Amblymelanoplia* spec. nov (Dombrow, 2002). (Photo credit: Mike Picker).

Female monkey beetles exhibit contrasting feeding patterns, which would determine their availability and apparency to males. Here we investigate how differences in feeding guild have driven the evolution of sexual dimorphism in male monkey beetles. A notable feature of South African monkey beetles is their array of leg weaponry size and shape (Figs. 1 and 2) and associated fighting behaviour, which appears to be a parallel of the extreme and bizarre array of head and thoracic horn development of Scarabaeoidea (dung beetles, fruit chafers, rhino beetles, and stag beetles) (Emlen et al., 2005; Moczek, 2005).

During the brief mass spring flowering event in the South African Cape region (Cowling, Esler & Rundel, 1999), disc- and bowl-shaped flowers of various species are focal points for male and female monkey beetle feeding and mating (Picker & Midgley, 1996; Goldblatt, Bernhardt & Manning, 1998; Goldblatt & Manning, 2011). Females of certain species embed and feed for extended periods within flower heads (the capitulum of Asteraceae or hypanthium of Aizoaceae). In contrast, males and females of the more active non-embedding guild forage briefly on pollen and nectar of widely dispersed flowers much like bees (Picker & Midgley, 1996). Females of both guilds lay their eggs in the soil (J. F. Colville, 2009, unpublished data). Females of the embedding guild theoretically provide a clumped and economically defendable reproductive resource for males, in contrast to the non-embedding species, where there would be fewer opportunities for female monopolisation.

**Figure 3:**
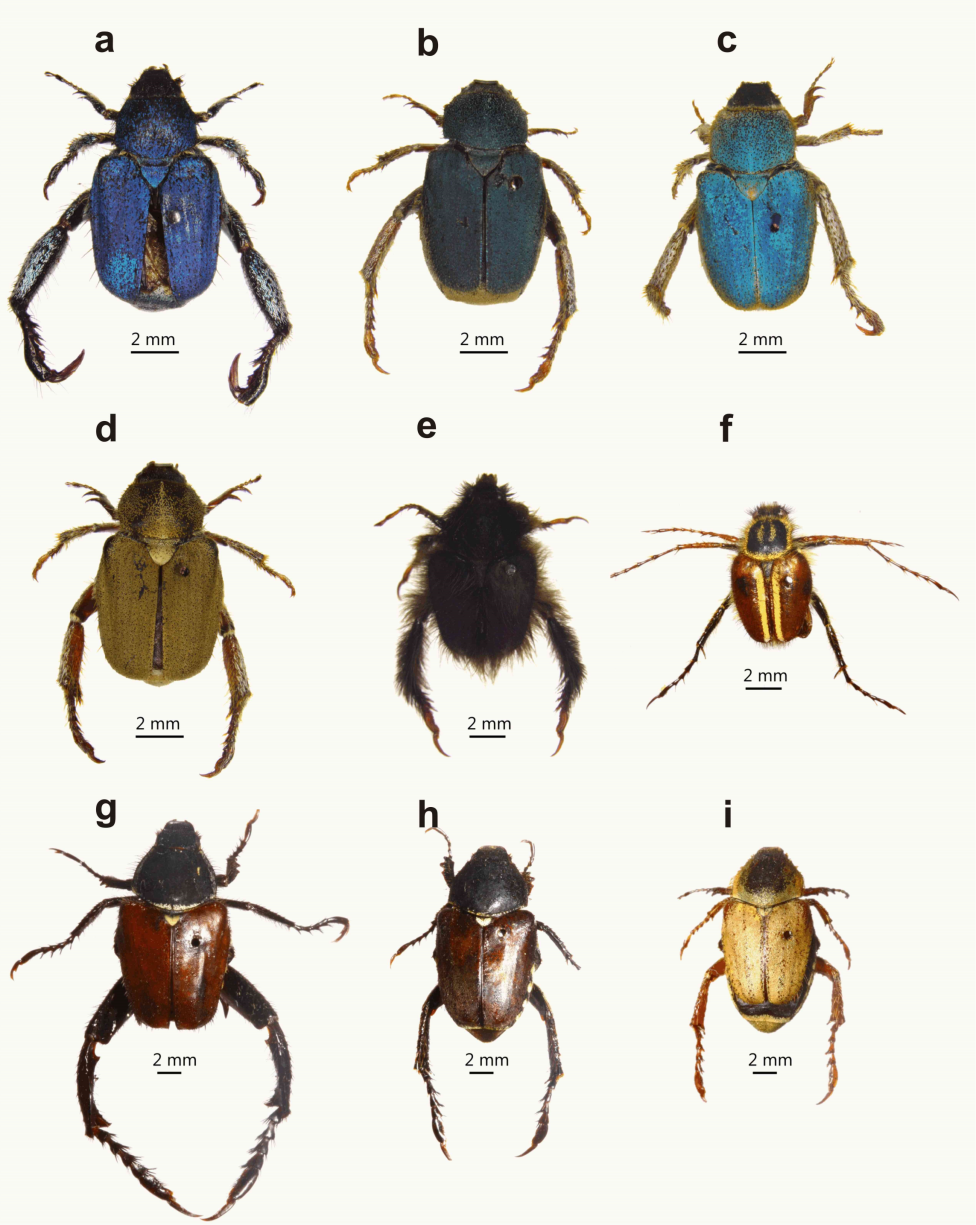
Examples of female colour polymorphism and non-dimorphic monkey beetles. Monkey beetle species showing female colour polymorphism ((A) male *Scelophysa trimeni* (Péringuey, 1902), (B–D) female *S. trimeni*; (G) male *Mauromecistoplia nieuwoudtvillensis* (Dombrow, 2002), (H and I) female *M. nieuwoudtvillensis*)) and examples of non-dimorphic species ((E) male *Peritrichia cinerea* (Olivier, 1789); (F) male *Lepithrix lineata* (Fabricius, 1775)). Colour polymorphism of females of *M. nieuwoudtvillensis* has one form (H) resembling the colour patterns of males and with the body not covered in scales; the other (I) matching the orange/yellow colour of its host plant with the body densely covered in scales. (Photo credit: Mike Picker).

While males of both guilds are polygamous, mating with multiple mates (M. Lewis, 2007, unpublished data), they differ in the extent of their hind leg weaponry and degree of sexual dimorphism. Embedders typically display hind leg and body colour dimorphism (Figs. 1 and 2), the former absent or weakly developed in non-embedders (Fig. 3). Male hind leg modification is expressed in hypertrophied leg components, changes in shape, and spine development. The modified legs are used as weapons (*sensu* McCullough, Miller & Emlen, 2016) in combat with rival males during mating and mate guarding (Louw, 1987; Midgley, 1992; Picker & Midgley, 1996). In monkey beetles, mate guarding (post copulation contact with females; Thornhill & Alcock, 1983) entails a male riding on a female’s back attached using his fore and midlegs; somewhat similar to that described for *Macrodactylus* (Macrodactylini) chafer beetles (Eberhard, 1993). Many male monkey beetle species of both guilds are brightly coloured, with duller, cryptically coloured females (Fig. 3). The role of colour in sexual selection is well established, acting either through female choice and/or as an indication of strength to rival males (Andersson, 1994). In monkey beetles, female choice does not appear to be operational, with females accepting multiple mates over short periods (M. Lewis, 2007, unpublished). Colour in males of both feeding guilds would broadly advertise the presence of a male to potential male competitors, obviating the need for combat (Outomuro, Adams & Johansson, 2013) and possibly reducing time spent searching for potential mates. In the case of embedders, the spatially fixed copulating and mate guarding beetles provide suitable opportunities for combat and takeover bids using the hind legs, and selection pressure for the evolution of leg weaponry (Emlen, 2008; McCullough, Miller & Emlen, 2016). Buried females often expose only a fraction of the pygidium (last dorsal segment of the abdomen) (Péringuey, 1902); possibly reducing the number of females readily visible to males, further skewing the operational sex ratio in favour of males, resulting in more intense competition for mates. The two published studies which have assessed the operational sex ratio in populations of monkey beetle have reported a male bias (Louw, 1987; Midgley, 1992); however, it is unclear if this is a general pattern across populations and not a sampling artefact. In contrast to embedders, male combat in non-embedders involves brief tussles between several males with limited mate guarding (Picker & Midgley, 1996). The lengthy mate guarding extends opportunities for male–male combat, but at the same time introduces the possibility of female sexual conflict (Chapman et al., 2003).

Several predictions emerge concerning the relationship between feeding ecology and sexual dimorphism among monkey beetles (Midgley, 1992). Females of sedentary, embedded species would provide opportunities for males to economically defend females (high mate monopolisation potential; Thornhill & Alcock, 1983), driving the evolution of male combat and weaponry, and positive allometric scaling relationships. In contrast, non-embedder females offer only brief matings (via scramble competition; Thornhill & Alcock, 1983) and limited mate-guarding opportunities, with reduced possibilities for male competition for females, and weaker selection for sexual dimorphism in leg weaponry. Bright colours would initially benefit males of both feeding guilds, as this would limit unprofitable contact with potentially competing males. For embedding species with sessile females, the opportunities for takeover bids would favour the additional evolution of weaponry.

Here we test these predictions by quantifying dimorphism scores based on hind leg morphology and body colour for a large number of monkey beetles (>360 species) belonging to the two contrasting feeding guilds. We also assess measures of allometric scaling relationships for males to identify the intensity of sexual selection on different hind leg traits. We discuss the relative roles of natural and sexual selection in the evolution of monkey beetle sexual dimorphism in the floristically diverse Cape region.

## Materials and Methods

### Sexual dimorphism

Beetles were classed as embedders or non-embedders using field observations and published information (Picker & Midgley, 1996; Goldblatt, Bernhardt & Manning, 1998; Steiner, 1998a, 1998b; Goldblatt & Manning, 2011). The extent of sexual dimorphism was compared in these two groups by quantifying hind leg, and body colour and pattern dimorphism (hereafter ‘colour dimorphism’). The degree of sexual dimorphism in leg morphology and body colouration between males and females was quantified on a point scoring system using 5,588 individuals (Table S1.1), representing over 371 species (∼31% of global species) and 42 South African genera (∼35% of global genera). This included species and genera from all parts of South Africa. Data were drawn from specimens in the collection of the Iziko Museums, Cape Town, South Africa and supplemented with field-collected material (CapeNature (Western Cape Province) permit numbers: AAA004-00530-0035, AAA007-00027-0056, AAA007-00081-0056, AAA007-00010-0056; Department of Environmental and Nature Conservation (Northern Cape Province) permit numbers: FAUNA 704/2009, FAUNA 761/2011, FAUNA 592/2012, FAUNA 2116/2015). Male and female pairs were selected haphazardly (i.e. in the order that they were found in a unit tray in a museum collection draw) from a series of beetles collected from a locality, and where possible, all available specimens for a species across all its collection localities were used in the analyses (Tables S1.1).

**Figure 4:**
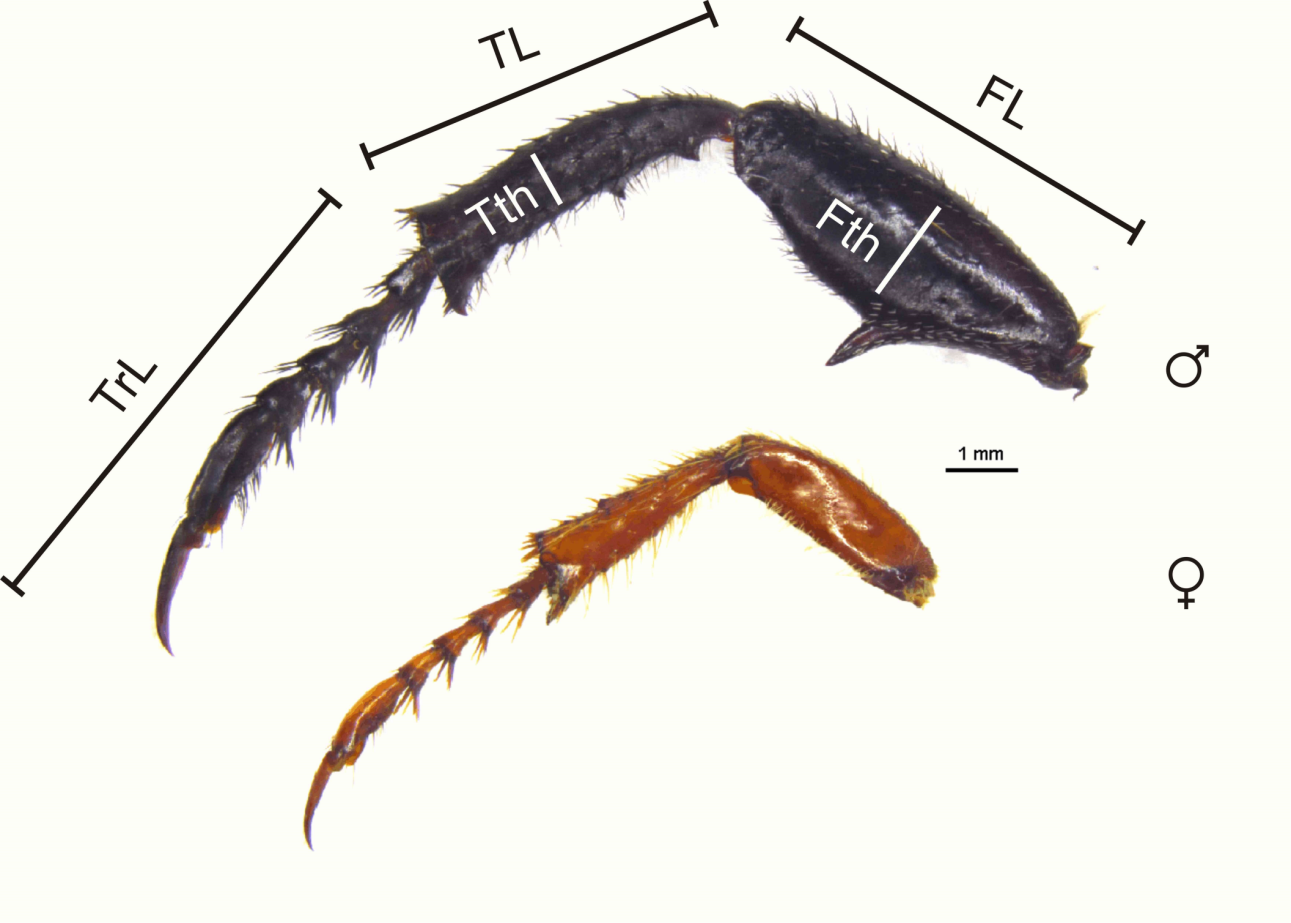
Male and female hind legs showing dimorphism scores. Male and female hind legs of *M. nieuwoudtvillensis* showing strong dimorphism (score of 6: 1 (femur longer) + 1 (femur thicker) + 1 (femur with spine) + 1 (tibia longer) + 1 (tibia thicker) + 1 (tibia with spine) + 0 (tarsi equal length) + 0 (no tarsal claw). FL, femur length, TL, tibial length, TrL, tarsal length, Fth, femoral thickness, Tth, tibial thickness. (Photo credit: Mike Picker).

Hind leg dimorphism scores were based on a three point scale for each hind leg segment, excluding the coxa which is small in these beetles (Fig. 4). Segment width and length were each evaluated on scores of 0 or 1; for each character a score of 1 indicated an exaggerated state in the male compared to the female, and a score of 0 indicated no difference between the sexes. In addition, scores of 1 or 0 indicated the presence or absence of femoral/tibial spines, and presence or absence of an enlarged tarsal claw (Tables S1.1 and S1.2). The scores for each hind leg segment were then summed to give a maximum hind leg score of nine. For length and width contrasts, a score of 1 indicated differences of at least 25% between male and female measurements (body size is similar in males and females). This percentage difference was arbitrarily selected; however, some guidance in choosing this was taken from Eberhard’s (1993; Table 1) study of secondary sexual characters associated with the fore, mid, and hind legs in species of Macrodactylini (the putative sister tribe of monkey beetles; Ahrens, Scott & Vogler, 2011), with males having on average 15.64% (±3.43) longer legs than females across species (see also Table 1 in Zeh, Zeh & Tavakilian, 1992; and Table 1 in Tseng & Rowe, 1999). All body measurements were taken with electronic digital callipers accurate to 0.01 mm.

**Table 1:** Numbers of species showing hind leg and colour dimorphism in the two feeding guilds.

(a) Hind leg dimorphism	Femur thicker (%)	Femur longer (%)	Femur spine (%)	Tibia thicker (%)	Tibia longer (%)	Tibia spine (%)	Tarsus thicker (%)	Tarsus longer (%)	Tarsal claw (%)
No. of embedders with:	149 (57.10)	204 (78.16)	81 (31.03)	161 (61.70)	227 (87.00)	97 (37.17)	164 (62.84)	239 (91.57)	62 (23.75)
No. of non-embedders with:	0	21 (19.10)	0	18 (16.36)	25 (22.73)	0	4 (3.64)	34 (30.91)	1 (0.91)

(b) Colour Dimorphism	Pronotum (%)	Elytra (%)	Pygidium (%)	Abdomen (%)					
No. of embedders with:	41 (15.71)	139 (53.26)	212 (81.23)	135 (51.72)					
No. of non-embedders with:	17 (15.45)	62 (56.34)	79 (71.82)	50 (45.46)					

Colour and pattern sexual dimorphism was measured on a binary scale. Total scores were based on the sum of scores from four body parts (pronotum, elytra, abdomen, and pygidium) where 0 was assigned to cases where males and females had the same colour and pattern of a body part, and 1 where they differed, providing a single value of 0 (monomorphic) to 4 (maximal dimorphism). Only differences in pure colour and pattern (not hue variations of the six primary colours) scored a 1, where hue variations were collapsed into one of the six ‘pure’ colours. Colour contrasts between the sexes were clear using these criteria, e.g. in Fig. 1, for instance, only species *k* and *l* achieved a score of 1 for elytra colour dimorphism. This was done in combination with pattern differences, so either (or both) colour and pattern differences could score a 1. Where colour polymorphism occurred in a species (Fig. 3) from a single locality, males and females were compared sequentially in the order in which they occurred in the series, and a median colour score generated. While some Monkey beetles may detect UV, the majority pollinate longer wavelength-emitting flowers (yellow to red). In trials done to detect their colour preferences, many species chose pigments with little UV reflectance (Picker & Midgley, 1996; Van Kleunen et al., 2007). Dafni et al. (1990) found that colour sensitivity in another flower-pollinating scarab (Glaphyridae) was optimised in the red spectral region. Given the lack of knowledge on monkey beetle vision, we could not assume a generality of UV reflection and detection. Colour in monkey beetles is produced by physical means (diffraction grids) and is thus fairly stable, i.e., specimens collected many decades ago retain their colours, as do specimens killed in ethyl acetate or cyanide. For the purposes of quantifying hue, spectrophotometric analysis would have provided more quantifiable and detailed results (Van Gossum et al., 2011), but we used a single observer to score differences in pure colour, given the very large number (>5,500) of specimens and species that were examined.

We examined the effect of guild on leg and colour scores using linear mixed effect models. As currently there is no comprehensive phylogeny available for the Hopliini, we were not able to utilise phylogenetic comparative analyses that take into account tree topologies and branch lengths (Felsenstein, 1985; Garland, Harvey & Ives, 1992; Martins & Hansen, 1997; Stone, Nee & Felsenstein, 2011). Species cannot be considered evolutionary independent data points in statistical analysis; the non-independence reduces degrees of freedom for hypothesis testing, lowers statistical power, and affects parameter estimation (Garland, Harvey & Ives, 1992; Stone, Nee & Felsenstein, 2011). We therefore followed the approach recently used by McCullough et al. (2015) where dimorphism in male rhinoceros beetle horns was assessed using taxonomy to account for shared evolutionary history. As several studies have highlighted, incorporating taxonomy is preferable to not having any measure of evolutionary history (Felsenstein, 1985; and see references in McCullough et al., 2015). We set genus as a random effect within the lme4 function (Bates et al., 2015) in R (version 3.4.1; R Development Core Team, 2017). Visual inspection of residual plots did not reveal any obvious deviations from homoscedasticity or normality. Significance values of the effect of guild were obtained by likelihood ratio tests of the full model with the effect in question against the model without the effect in question (Bates et al., 2015).

### Hind leg scaling relationships

Scaling relationships (Shingleton et al., 2007) for hind leg lengths and widths of males from 37 species (minimum of 20 individuals per species; Table S2) were described based on slopes estimated from major axis (MA) regression (Warton et al., 2006) between body length (front part of the head (clypeus) to tip of pygidium) and trait size (both log transformed; Table S3) using the R package ‘smart’ (Warton et al., 2012). As for dimorphism scores above, we used linear mixed effect models to test if feeding guild (set as a fixed effect) had an effect on slope values, with genus set as a random effect. Significance values were obtained by likelihood ratio tests where the full model including the effect of guild was compared against an intercept only model.

## Results

### Sexual dimorphism

#### Hind legs

**Figure 5:**
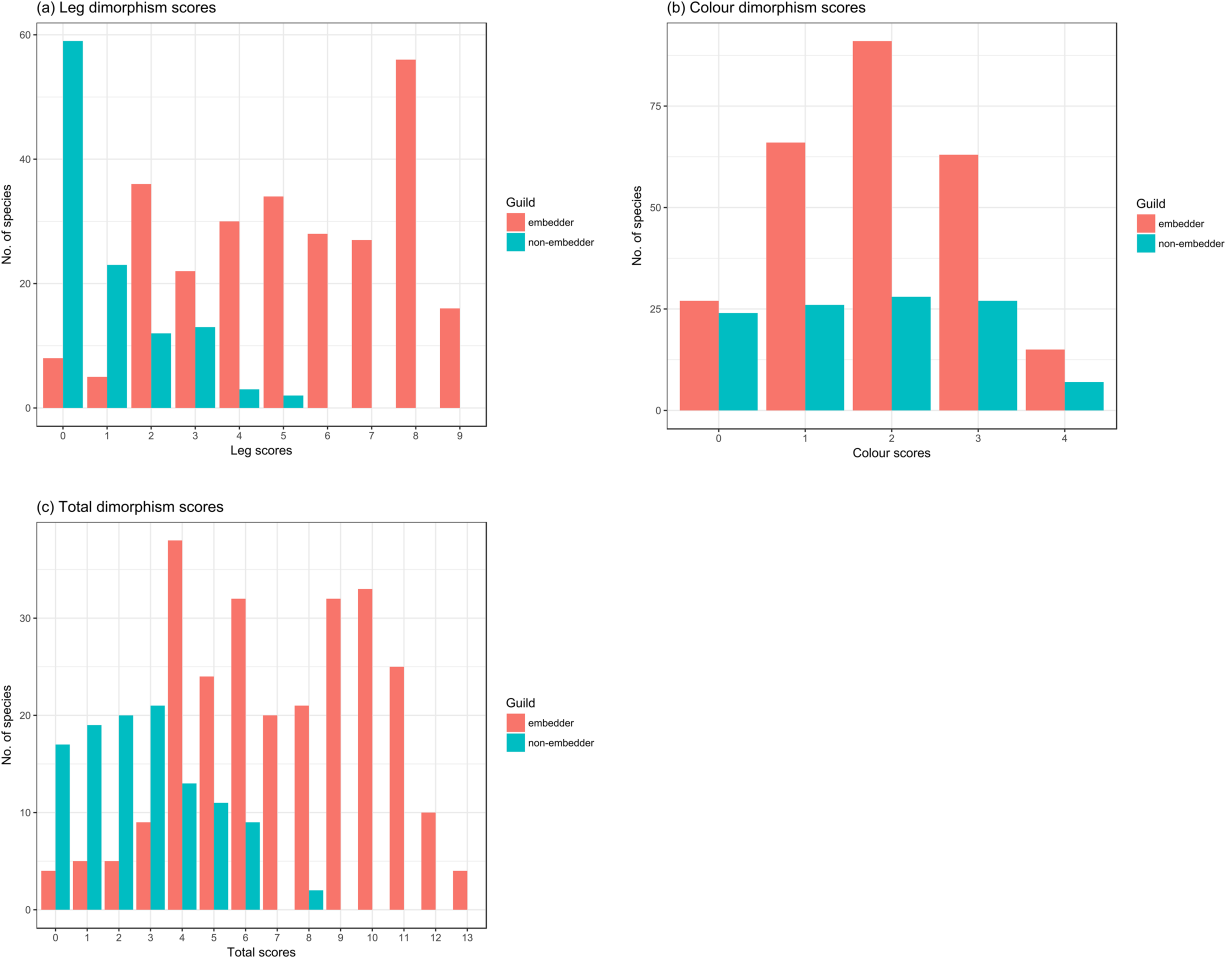
Counts of species from the two different feeding guilds for dimorphism scores for leg and colour. Counts of embedder and non-embedder species showing respective dimorphism scores for (A) leg, (B) colour, and (C) total score. High scores represent strongly sexually dimorphic species.

Hind leg dimorphism scores ranged from 1 to 9 across guilds (Fig. 5A; Table S1.2) and were mostly reflected in thicknesses and lengths of the femur, tibia, and tarsus (Table 1). Tarsal length was a dimorphic hind leg feature in a large number of species (72% of all species scored). Tibial (24.3%) and femoral spines (22.4%), and modified tarsal claws (17%) were less common, generally only noted in species with highest hind leg dimorphism scores. Guild type had a significant effect on hind leg dimorphism scores (χ^2^(1) = 28.39, *P* < 0.0001) with non-embedders having lower leg scores (*b* = 3.94 ± 0.61 (SE), *t* = 6.46, *P* < 0.0001) compared to those of embedders. Non-embedders mostly had weak hind leg dimorphism, seen in femoral, tibial, and tarsal lengths. In sharp contrast to embedders, no non-embedders showed femoral thickness dimorphism, and only 18.2% of non-embedders showed dimorphism in tibial thickness. As in embedders, the tibia appeared to be the most dimorphic hind leg segment (Table 1). Tibial and femoral spines, and modified tarsal claws were almost exclusively features of embedders with only a single non-embedder showing tarsal claw dimorphism (*Peritrichia nuda* (Schein, 1959)).

#### Colour

Colour dimorphism was widespread across both guilds with a high proportion of species (82.7%) showing colour dimorphism in at least one of the scored body parts (Fig. 5B), particularly the pygidium (73.62%), followed by the elytra (48.24%) (Table 1). In contrast to leg scores, guild had no significant effect on colour dimorphism (χ^2^(1) = 0.71, *P* > 0.1) with scores of non-embedders only marginally lower (*b* = 0.24 ± 0.28 (SE), *t* = 0.85, *P* > 0.1) to those of embedders.

### Hind leg scaling relationships

**Figure 6:**
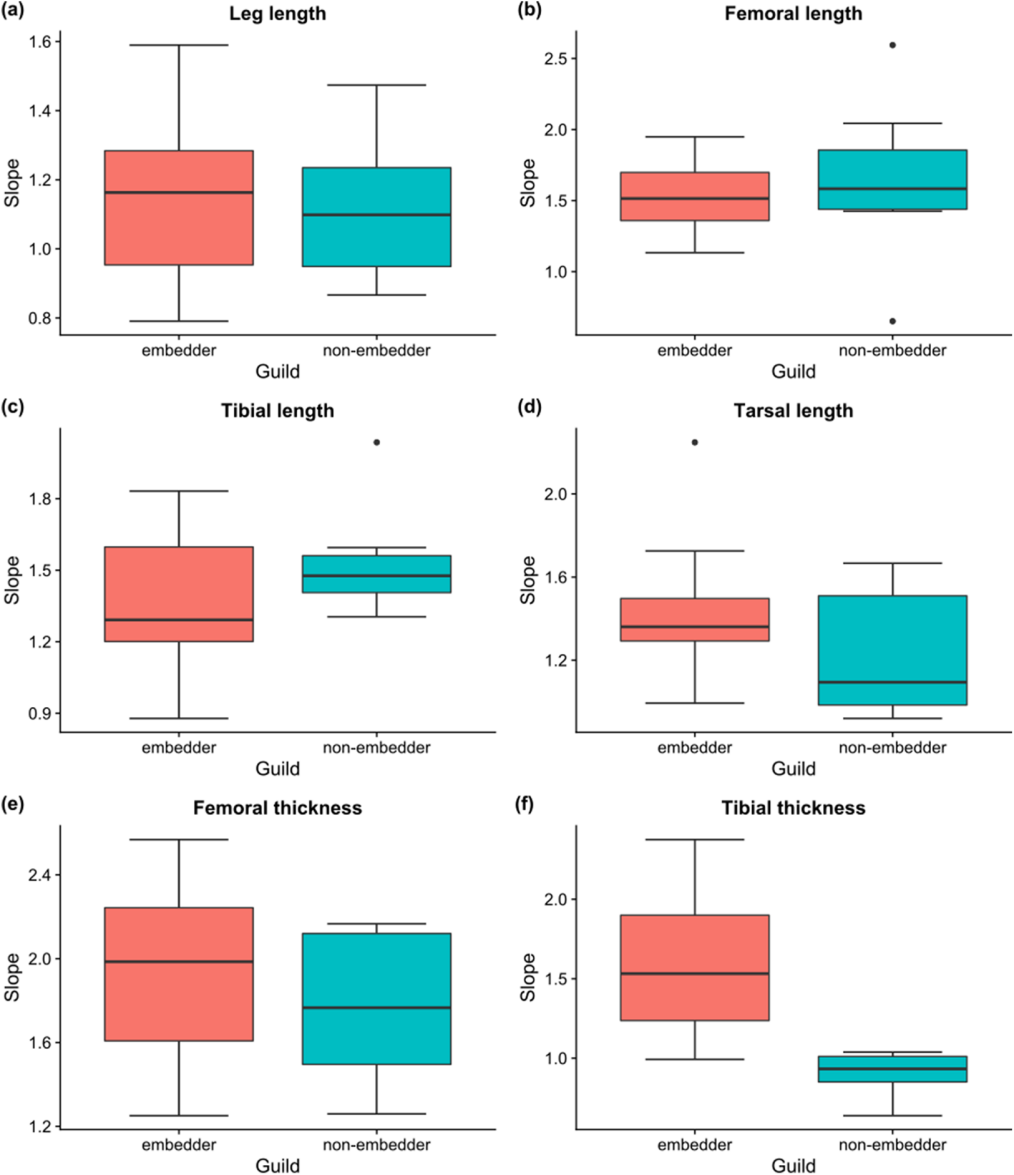
Boxplots of slopes of allometric relationships for hind leg traits for different feeding guilds. Boxplots of slopes (*b*_MA_) of allometric relationships for hind leg traits for embedders and non-embedders: (A) leg length, (B) femoral length, (C) tibial length, (D) tarsal length, (E) femoral thickness, (F) tibial thickness. Solid horizontal line indicates the median, and the lower and upper hinges correspond to the first and third quartiles, while the upper whisker extends to the largest value no further than 1.5 times the IQR (inter-quartile range), and the lower whisker extends to the smallest value 1.5 times IQR.

Linear allometric relationships between body length and hind leg trait size were recorded for the vast majority of species, with ∼83% of male embedders and ∼47% of male non-embedders showing significant (*P* < 0.05) scaling relationships between body length and hind leg trait sizes (averaged across all six hind leg traits) (Table 2). Allometric slopes of 1.50–1.70 were most common for male embedders, with the steepest allometric slopes (*b*_MA_ > 2.0) seen for femoral thickness (10 species) and femoral length (*b*_MA_ > 1.5; nine species) (Fig. 6). Highest recorded slopes were observed for femoral thickness in the two embedders *Denticnema decemlineata* (Dombrow, 1997) (*b*_MA_ = 2.57) and *Pachycnema crassipes* (Fabricius, 1775) (*b*_MA_ = 2.34). A high percentage of embedders (>70%) showed positive allometry for femoral length, femoral thickness, tarsal length, tibial length, and tibial thickness (Table 2), whereas fewer non-embedders showed positive allometries (∼40%) for these traits. Negative allometry was the exception, occurring for leg length in ∼27% of embedder species. However, for several of these species, leg length slopes were close to one suggesting a possible isometric relationship.

**Table 2:** Percentages of embedders and non-embedders showing positive and negative allometric relationships.

	FL	FTh	LL	TrL	TL	TTh	Average (±SD)
E: *b*_MA_ > 1	81.82	90.91	63.64	72.73	77.27	72.73	76.55 (9.28)
*b*_MA_ < 1	–	–	27.23	4.55	4.55	4.55	6.82 (10.27)
NE: *b*_MA_ > 1	53.33	46.67	40.00	20.00	46.67	13.33	36.67 (16.19)
*b*_MA_ < 1	6.67	–	20.00	13.33	–	26.67	11.11 (10.87)

**Notes:**

Percentages of embedders (22 species) and non-embedders (15 species) showing positive (*b*_MA_ > 1) and negative (*b*_MA_ < 1) allometric relationships between hind leg trait size and body length. Trait percentages do not add up to 100%; remaining percentage is reflective of those species showing a non-significant relationship.

Trait percentages for: LL, leg length; FL, femoral length; TL, tibial length; TrL, tarsal length; TTh, tibial thickness; FTh, femoral thickness; E, embedder; NE, non-embedder.

For male non-embedders ∼53% showed slopes of *b*_MA_ > 1 for femoral length. Approximately 30% of the remaining leg traits for non-embedders showed positive allometric slopes (Table 2), mostly falling between 1.2 and 1.8 (Fig. 6) and femoral length and femoral thickness showed the steepest allometric slopes (*b*_MA_ > 2.0). Overall, embedders generally had steeper allometric slopes across leg traits (apart from femur and tibia length) when compared to non-embedders (Fig. 6). Overall, only for tibial thickness did feeding guild have an effect on slope (χ^2^(1) = 12.80, *P* > 0.001), with non-embedders having significantly lower slope values (*b* = 0.66 ± 0.56, *t*_23_ = 4.14, *P* < 0.001).

## Discussion

### Feeding ecology and sexual selection

The intensity of sexual selection is related to the availability of the limiting sex (normally females), the latter related to ecological context (Emlen & Oring, 1977; Thornhill & Alcock, 1983; Shuster, 2009). For ecological context to influence sexual selection there needs to be a predictable spatial distribution of a resource that is attractive to females (resource defence), or females themselves (female defence), and where it is economically profitable for males to compete for these (Emlen & Oring, 1977; Thornhill & Alcock, 1983). Male beetles from several different families defend localised sap fluxes, visited by females for feeding and mating (Snell-Rood & Moczek, 2013). Thornhill & Alcock (1983) list several examples of resource defence in other insects, such as male bees defending flower patches, male coreid bugs defending plant stalks, and male dragonflies defending oviposition sites. Examples of female defence are recorded for different weevil species, Japanese flower beetles, and tenebrionid ground beetles (Thornhill & Alcock, 1983; Snell-Rood & Moczek, 2013). Many of these systems include alternative mating strategies for males that do not defend fixed resources or females (Gross, 1996; Emlen, 2008; Snell-Rood & Moczek, 2013; Buzatto & Machado, 2014).

Monkey beetle feeding guilds provide interesting ecological contrasts in the spatial predictability of females, with the embedding guild theoretically representing a fixed and economically defendable reproductive resource for males, with the dispersed females of the non-embedding guild representing a spatially unpredictable resource (Midgley, 1992). The embedding behaviour would favour the evolution of male competition and leg weaponry, and predictably we found that hind leg dimorphism was largely restricted to this guild (∼87% of species examined). Species in this guild exhibited hind leg dimorphism in all leg parts, typically having femur and tibia swollen with exaggerated musculature and armed with combinations of spines, recurved teeth, and spurs. In some species, enlarged tarsi comprised *∼*74% of the entire body length (e.g. *Heterochelus bivittatus*) (Burmeister, 1844) (Fig. 1). Non-embedders showed weak hind leg dimorphism (∼8% of examined species) and lacked the extensive range of weaponry of embedders (e.g. tibial and femoral spines, and modified tarsal claws), apart from an elongation of the tibia and tarsus, sometimes accompanied by extensive pilosity, forming tarsal or tibial ‘socks’—a feature that appears unique to non-embedders. The sessile and defendable distribution of females on flowers, possibly combined with a stable platform for fighting (the flat disk shape of host flowers), may provide opportunity for embedder males with relatively larger and more elaborate hind leg weapons (spines, spurs, etc.) to be competitively superior during contests. This hypothesis is supported by the reduction of male hind leg dimorphism in non-embedding species, which have less physically aggressive male contests, possibly also influenced by the unsuitability of non-disk-shaped host flowers as stable platforms for contests, and less localised distributions of females. The most speciose genera in the two feeding guilds exemplify this pattern—embedding *Heterochelus* (195 species) had 93.8% of species scored as hind leg dimorphic; in contrast to the 12.5% for non-embedding *Peritrichia* (Burmeister, 1844) (74 species).

The extensive development of hind leg dimorphism in South African monkey beetles appears to represent a parallel variation of weaponry and associated sexual selection in Scarabaeoidea where cephalic and thoracic horns are employed as weapons (Emlen & Nijhout, 2000; Emlen et al., 2005; Moczek, 2005; Emlen, 2008). Hind leg dimorphism of monkey beetles is somewhat analogous to the leg dimorphism recorded for some leaf beetles (Chrysomelidae) (Eberhard & Marin, 1996; Katsuki et al., 2014) and twig-wilter bugs (Coreidae) (Miyatake, 1997; Eberhard, 1998), where males use their hind legs as weapons in combat over females, and where the hind legs are also subject to sexual selection.

### Colour dimorphism

Colour dimorphism was evident in 85% of species, more common to embedders (68% of examined species) than non-embedder guild members (31% of examined species). The most extreme form of colour dimorphism included complete colour dichromatism in all body parts, (Fig. 3), generated by variations in the pattern and density of cuticular scales. The most common colour dimorphic body parts were the pygidia and elytra, with the former showing dimorphism in ∼70% of species. The pygidium is often uniquely coloured compared to the rest of the body, and commonly showed both morphological and colour differences between males and females, especially amongst embedders. Male pygidia are noticeably larger than those of females, and strikingly marked with contrasting patterns of white, yellow, orange, and black scales. The pygidium is often the only body part of a male embedder exposed when mating and guarding on the flower disc, suggestive of male–male signalling. In contrast, female pygidia are typically uni-coloured and commonly match the colour of host disc florets. Whether this is an adaptation for predator avoidance or possibly related to sexual conflict (Chapman et al., 2003) is not known. The presence of male-mimicking female colour morphs (andromorphs) (Fig. 3) suggests that sexual conflict may be at play (Van Gossum, Stoks & Bruyn, 2004). Colour dimorphism with brightly coloured males and cryptic females is typically associated with either mate attraction, or with territoriality and advertisement to potentially competing males during copulation (Andersson, 1994; Berglund, Bisazza & Pilastro, 1996). Monkey beetles have acute vision, and are able to differentiate reflectance spectra, patterns, and hue, largely in the longer wavelength spectra (Midgley, 1993; Picker & Midgley, 1996; Steiner, 1998a; Van Kleunen et al., 2007).

In monkey beetles there apparently is no role for female choice (Louw, 1987; Midgley, 1992; M. Lewis, 2007, unpublished data), and the physical cost of male contests in monkey beetles is potentially high due to physical injury. With the diverse armaments of large spines and cutting edges (Figs. 1 and 2) embedder males have the ability to amputate another male’s leg during combat. We have observed male embedders missing entire hind legs or distal parts of the hind leg, as well as some males with the severed leg component of a competitor still clamped within their hind femoral-tibial joint. Colour in males (especially of a weapon) would be an expected parallel adaptation to the weapons themselves, allowing males searching for females the option of assessing or avoiding other males that are engaged in copula or guarding females (Moore, 1957; Emlen, 2008; Tedore & Johnsen, 2012). Male embedders often raise their hind legs prominently during copulation and mate guarding. The hind legs would appear to function as both weapons and ornaments in male–male assessment (Emlen, 2008; McCullough, Miller & Emlen, 2016).

### Sexual selection and scaling relationships in hind legs

Monkey beetles with highly dimorphic traits had allometric slopes comparable to those recorded for other insect groups undergoing strong sexual selection on male traits (Kodric-Brown, Sibly & Brown, 2006). In contrast, non-embedders which appear to experience less intense sexual selection (at least on hind leg traits) generally showed shallower slopes and no significant relationships between body size and trait size. Exaggerated traits used in male combat which display positive allometric slopes (*b*_MA_ > 1) are mostly indicative of directional sexual selection (Andersson, 1994; Emlen & Nijhout, 2000; Bonduriansky & Day, 2003; Kodric-Brown, Sibly & Brown, 2006). For many insects, the shape of the relationship between body size and trait size can deviate from linearity (Eberhard & Gutierrez, 1991; Emlen & Nijhout, 2000; Pomfret & Knell, 2006; Kodric-Brown, Sibly & Brown, 2006), as allometric slopes in insects are strongly influenced by environmental factors (Emlen, 1994), the extent of intrasexual selection (Kodric-Brown, Sibly & Brown, 2006; Bonduriansky, 2007) and functional constraints on body parts imposed by habitat and diet (Fairbairn, 1997). Non-linear or sigmoid allometric relationships have also been shown to be reflective of alternative mating strategies in contrasting morphs, i.e., within a species large males with large traits (major morph) aggressively defend females or resources, whereas small males (minor morphs) lacking such weapons are non-aggressive and rather use alternative mating strategies, such as sneak mating (Thornhill & Alcock, 1983; Emlen & Nijhout, 2000; Moczek, 2005; Karino, Niiyama & Chiba, 2005; Buzatto & Machado, 2014). In monkey beetles, exaggerated hind leg traits are expressed in both large and relatively smaller sized males—i.e., absence of distinctive major and minor morphs. Therefore, in monkey beetles it appears as if only a single mating strategy is operational within a species.

Usually a single leg component (e.g., the femur) showed positive allometry, but sometimes more than one leg component (femur + tibia) showed this relationship. This suggests that different fighting behaviours might be used. For example, both *Pachycnema crassipes* (Fabricius, 1775) (Fig. 1) and *Pachycnema alternans* (Burmeister, 1844) (Fig. 2) have unusually round and swollen tibiae (tibial thickness slopes of *b*_MA_ > 1.5), whereas in *Pachycnema calcarata* (Burmeister, 1844) the femora are especially well-developed (femoral thickness *b*_MA_ = 2.33). However, almost nothing is known about the various species-specific fighting behaviours in monkey beetles, and how these relate to exaggerated hind leg shape and size and placement of spines, or the mechanical strength of the many different shapes and sizes of hind leg weapons. Detailed data are required on male–male combat, shape and size variations, and biomechanical measurements to understand why some hind leg traits have higher scaling relationships. Understanding the selective processes underlying the divergence of male weaponry in monkey beetles lags far behind that of other scarab groups (Emlen et al., 2005; Snell-Rood & Moczek, 2013), in spite of the widespread and conspicuous male weaponry of the group. Studies undertaken for rhinoceros (McCullough, 2014; McCullough, Tobalske & Emlen, 2014) and stag beetles (Goyens et al., 2014; Goyens, Dirckx & Aerts, 2015a, 2015b) are leading the way in methodological techniques for measuring the structural and biomechanical strengths of male weaponry.

### Origins of monkey beetle dimorphism and diversity

Monkey beetles (Hopliini: Scarabaeidae) show exceptional diversity in the Cape region, which contains >85% of the South African monkey beetle fauna (>500 species), and ∼50% of the world’s fauna, highlighting the importance of this region as a global centre of radiation of many taxa (Colville et al., 2014). Various factors have been proposed for the extensive radiation of insect groups in the region; including coevolution with host plants (Colville et al., 2014), specialised adaptations for exploiting the diverse floral resources (Stuckenberg, 2000; Karolyi et al., 2014, 2016), high landscape and environmental heterogeneity (Colville, Picker & Cowling, 2002), and long-term climatic stability (Pitzalis & Bologna, 2010). Sexual selection may also have had a role in promoting monkey beetle diversity, as has been proposed for other diverse taxa, e.g., the explosive and rapid speciation of Hawaiian insects is thought to have been strongly driven by sexual selection (Mendelson & Shaw, 2005; Kaneshiro, 2006; O’Grady, Magnacca & Lapoint, 2010). The majority of the 371 species of Hopliini examined showed sexual dimorphism, with 64% of species showing hind leg dimorphism, with almost two-thirds of examined species being sexually dimorphic for both colour and hind legs. However, to evaluate if sexual selection is a driver of speciation in monkey beetles, a comprehensive phylogeny is required to enable clade comparisons and track the evolution of feedings patterns and the diversity of hind leg weapons across different monkey beetle habitats (Emlen et al., 2005; Freckleton & Jetz, 2009). Contrasting sister clades for degree of sexual dimorphism and feeding guild membership would provide insight into the importance of sexual selection in diversification (Barraclough, Harvey & Nee, 1995; Panhuis et al., 2001; Gage et al., 2002; Morrow, Pitcher & Arnqvist, 2003; Okada et al., 2008; Seddon et al., 2013). There are currently, however, no detailed phylogenetic studies of South African monkey beetles, and those that do exist (Ahrens, Scott & Vogler, 2011; Carrillo-Ruiz & Morón, 2011) do not have a sufficiently dense sampling of taxa to allow for identification of discrete lineages and an interpretation of the evolution of sexual dimorphism in the South African Hopliini. In the absence of a resolved phylogeny for the tribe, it is not possible to speculate at this stage on the ancestral hind leg condition or whether hind leg dimorphism evolved multiple times in association with the evolution of different feeding ecologies. Our results of the effect of feeding guild on dimorphism should be treated with some caution; our analyses were limited in their ability to statistically control for the evolutionary relationships among taxa (see Felsenstein (1985) for discussion on the statistical limitations of using taxonomy to control for non-independence). Revealing the relationship between dimorphic traits across related taxa requires that any non-independence in the data, such as shared common ancestry, be accounted for statistically to control for type I and II error rates and overestimates of degrees of freedom (Stone, Nee & Felsenstein, 2011). A phylogenetic comparative method that explicitly considers evolutionary history would be needed to validate our results and to assess patterns of covariation among traits across species and assess the evolutionary causes of convergence in relation to feeding guild. As such, the ancestral states of traits in monkey beetles and their sequence of evolutionary change can only be speculated at this stage. The putative sister tribe of Hopliini viz. Macrodactylini (Ahrens, Scott & Vogler, 2011; Ahrens, Schwarzer & Vogler, 2014) shows some degree of leg, but not colour dimorphism (Eberhard, 1993; Mondaca & Federico, 2012). Extreme leg dimorphism was evident in the embedding guild, and it might therefore be hypothesised (given the condition in Macrodactylini), that the embedding habit was the ancestral feeding condition for the South African Hopliini, and that shifts to the non-embedding guild, with concomitant loss of hind leg dimorphism evolved subsequent to this. However, the tribe Hopliini apparently evolved in the Palaeocene ∼60 Mya (Ahrens, Schwarzer & Vogler, 2014) whereas their main host plants arose much later than this. Asteraceae only diversified and radiated in the Oligocene and Miocene in the Southern hemisphere (∼7–28 million years ago) (Barreda et al., 2010), and a major clade of the Aizoaceae (Ruschiodea) arose even more recently (3.8–8.7 Mya) (Klak, Reeves & Hedderson, 2004). Therefore the ancestral Hopliini likely utilised flowers of early diverging flowering plants or even their foliage. In the case of the monkey beetles, specialised associations with plant species within diverse floral niches likely initiated the radiation, which was then followed by the evolution of complex and divergent mating behaviours, including male–male combat, and sexual selection.

## Conclusion

This is the first study to document the patterns of sexual dimorphism in South African monkey beetles, and place these patterns in the context of contrasting mating behaviour across different feeding guilds. Species where females feed at a fixed resource predictably showed higher levels of sexual dimorphism and positive scaling relationships for male hind leg weaponry, suggestive of a high intensity of sexual selection. The degree of colour dimorphism between the sexes although extensive was not, however, associated with any particular feeding guild, and may serve as a generalised intrasexual signal between males to reduce physical conflict and combat. Understanding the selective processes underlying the evolution of sexual dimorphism in monkey beetles lags far behind that of other scarab groups (Emlen et al., 2005), in spite of the widespread and conspicuous male weaponry of the group. Improved phylogenetic data is required to better understand the relationship between feeding guild, sexual dimorphism and sexual selection in the radiation of the diverse monkey beetle fauna of Southern Africa.

## Supplemental Information

10.7717/peerj.4632/supp-1 Supplemental Information 1 Raw data–Leg and body colour scores. Raw data–Leg and body colour scores used to quantify the extent of sexual dimorphism in hind leg and body colour dimorphism for two feeding guilds (embedders and non-embedders). Pair # refers to the numbers of male-female comparisons assessed per locality (a collection series). For hind legs, a score of 1 indicated an exaggerated state in the male compared to the female, and a score of 0 indicated no difference between the sexes. In addition, scores of 1 or 0 indicated the presence or absence of femoral/tibial spines, and presence or absence of an enlarged tarsal claw. For colour scores, 0 was assigned to cases where males and females had the same colour and pattern of a body part, and 1 where they differed.

10.7717/peerj.4632/supp-2 Supplemental Information 2 Raw data–Mean leg and body colour scores. Raw data–Mean leg and body colour scores across collection localities for a species.

10.7717/peerj.4632/supp-3 Supplemental Information 3 Raw data–Scaling relationships for male hind leg lengths and thicknesses. Raw data–Calculation of scaling relationships for male hind leg lengths and thicknesses for 37 species (minimum of 20 individuals per species).

10.7717/peerj.4632/supp-4 Supplemental Information 4 Raw data–Coefficients of the fitted major axes regression models with slope and upper and lower confidence intervals (CI). Raw data–Coefficients of the fitted major axes regression models with slope and upper and lower confidence intervals (CI). Data from Table S2 were used in the regression models of male body lengths against hind leg traits (lengths and thicknesses).

## Additional Information and Declarations

### Field Study Permissions

The following information was supplied relating to field study approvals (i.e. approving body and any reference numbers): Field permits were received from CapeNature (Western Cape Province) and Department of Environmental and Nature Conservation (Northern Cape Province): AAA004-00530-0035, AAA007-00027-0056, AAA007-00081-0056, AAA007-00010-0056; FAUNA 704/2009, FAUNA 761/2011, FAUNA 592/2012, FAUNA 2116/2015.

### Data Availability

The following information was supplied regarding data availability: The raw data are provided in the Supplemental Files.
